# Regulation of dendrite morphology and excitatory synapse formation by zDHHC15

**DOI:** 10.1242/jcs.230052

**Published:** 2019-07-05

**Authors:** Bhavin S. Shah, Jordan J. Shimell, Shernaz X. Bamji

**Affiliations:** Department of Cellular & Physiological Sciences & the Brain Research Centre, University of British Columbia, 2350 Health Sciences Mall, Vancouver, B.C., V6T-1Z3, Canada

**Keywords:** zDHHC15, Palmitoyl transferases, Palmitoylation, Excitatory synapses, PSD-95, DLG4, Dendrite morphology

## Abstract

Protein palmitoylation is the most common post-translational lipid modification in the brain and is mediated by a family of 24 zDHHC enzymes. There has been growing interest in zDHHCs due to mounting evidence that these enzymes play key roles in the development and function of neuronal connections, and the fact that a number of zDHHCs have been associated with neurodevelopmental and neurodegenerative diseases. Loss-of-function variants in several zDHHCs, including zDHHC15, have been identified in patients with intellectual disabilities; however, the function of zDHHC15 in the brain has not been well studied. Here, we demonstrate that knocking down zDHHC15 in primary rat hippocampal cultures reduces dendritic outgrowth and arborization, as well as spine maturation. Moreover, knockdown of zDHHC15 reduces palmitoylation of PSD-95 and its trafficking into dendrites, resulting in an overall decrease in the density of excitatory synapses being formed onto mutant cells.

## Abstract (precis)

**Summary:** Disruption of zDHHC15 function in primary hippocampal cultures reduces dendritic outgrowth, spine maturation, excitatory synapse density and trafficking of PSD-95 into dendrites.

## INTRODUCTION

Post-translational modification of cellular proteins by S-acylation involves the reversible attachment of fatty acids to cysteine residues, and is important for the trafficking of proteins towards cell membranes and the regulation of cellular signaling (Fukata and Fukata, 2010; Ko and Dixon, 2018). Palmitoylation is the reversible attachment of the fatty acid palmitic acid, and is the most common post-translational lipid modification in the brain (Fukata and Fukata, 2010). Enzymes that mediate palmitoylation consist of a family of 23 proteins containing a conserved Asp-His-His-Cys (DHHC) motif that is required for enzymatic activity (Fukata and Fukata, 2010). Nine of the 23 genes encoding zinc finger DHHC-type (zDHHC) enzymes have been associated with brain disorders and ∼41% of all identified synaptic proteins are substrates for palmitoylation (Sanders et al., 2015), underscoring the importance of palmitoylation in the development and function of the brain and, specifically, in synapse biology. Despite their implied importance, little is known about the role of zDHHC enzymes in the brain and whether disrupting their enzymatic activity contributes to brain pathology (Charollais and Van Der Goot, 2009; Fukata and Fukata, 2010; Rocks et al., 2010).

Although zDHHC15 is highly expressed in many cell types in the brain (Fukata et al., 2004; Mansouri et al., 2005; Wang et al., 2015; Zhang et al., 2014), little is known about its function. Known substrates of zDHHC15 include PSD-95 (officially known as DLG4), GAP43, SNAP25b, CSP (officially known as DNAJC5), GABARγ2 (officially known as GABRG2) , Fyn, BACE1, CD151, CIMPR (officially known as IGF2R) and SORT1 (Fang et al., 2006; Fukata et al., 2004; Greaves et al., 2010, 2008; Mill et al., 2009; Sharma et al., 2008; Tsutsumi et al., 2009; Vetrivel et al., 2009; Yokoi et al., 2016), proteins that have been shown to play an important role in the development and function of neuronal connections. *ZDHHC15* is one of a number of genes on the X chromosome that is duplicated in patients with intellectual disability (Linhares et al., 2016; Martinez et al., 2014), and one report has identified the loss of a *ZDHHC15* transcript in a female patient with non-syndromic X-linked intellectual disability (Mansouri et al., 2005).

Our study characterizes the role of zDHHC15 in the development of neuronal connectivity. zDHHC15 is highly expressed at embryonic stages and reduced in adult brains. Knockdown of zDHHC15 in hippocampal cultures reduces dendritic arborization and spine maturation. Notably, zDHHC15 knockdown results in a marked reduction in the density of excitatory synapses, resulting from reduced palmitoylation of PSD-95 and its trafficking into dendrites.

## RESULTS AND DISCUSSION

### zDHHC15 is expressed during early stages of brain development, and in cultured excitatory and inhibitory hippocampal neurons

**Fig. 1.**
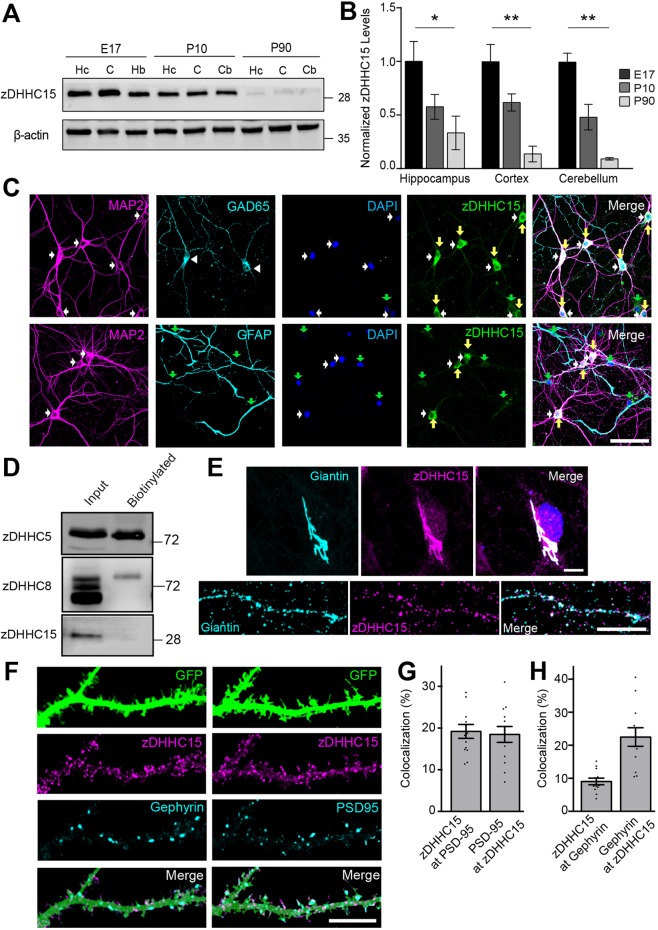
**zDHHC15 is expressed during early stages of neocortical development and in Golgi compartments of cultured excitatory and inhibitory neurons.** (A,B) Western blot and quantification of zDHHC15 protein levels in the hippocampus (Hc), cerebral cortex (C), hindbrain (Hb) or cerebellum (Cb) of mouse brain at E17, P10 and P90. Levels of zDHHC15 decreased from E17 to P90 in these regions of mouse brain. *n*=4 tissue samples per time point, *P<0.05, **P<0.001, one-way ANOVA with Tukey's post-hoc test, means±s.e.m. (C) Representative confocal images of 13 DIV rat hippocampal cultures. All neurons, identified by using anti-MAP2 antibody (white arrows), expressed zDHHC15 (yellow arrows) – including inhibitory GAD65-positive neurons (white arrowheads). GFAP-positive glial cells (green arrows) exhibited weak to no zDHHC15 immunostaining. White arrows denote DAPI-stained nuclei that are positive for zDHHC15, whereas green arrows denote DAPI-stained nuclei that are negative for zDHHC15. (D) Western blot of lysates from biotinylated neurons at 13 DIV probed for zDHHC5, zDHHC8 and zDHHC15; the latter is not localized at the membrane. *n*=3 cultures. (E) zDHHC15 colocalizes with the cis-medial Golgi marker giantin. (F-H) Microimages (F) showing 13 DIV rat hippocampal neurons stained for zDHHC15 and the synaptic markers PSD-95 and gephyrin. zDHHC15 colocalizes with a small subset of PSD-95 or gephyrin puncta. zDHHC15 and PSD-95 images were masked as per Fig. S2E. *n*=10 neurons, three cultures. Scale bars: 100 µm (C), 20 µm (E, top) and 10 μm (E, bottom; F).

*ZDHHC15* mRNA is abundantly expressed in the brain compared to other tissues (Fukata et al., 2004). After validating the specificity of our zDHHC15 antibody (Fig. S1A-C), we determined that zDHHC15 is highly expressed during early stages of development – i.e. at embryonic day 17 (E17) and postnatal day 10 (P10) – and significantly reduced in the adult (P90) brain (Fig. 1A,B).

To further examine the cellular and subcellular distribution of zDHHC15, we immunostained primary hippocampal cultures at 13 days *in vitro* (DIV). zDHHC15 was observed in all neurons (identified by using anti-MAP2 antibody) – including GAD65-positive inhibitory neurons, demonstrating that zDHHC15 is expressed in both excitatory and inhibitory neurons. In contrast, in these young cultures, zDHHC15 was not expressed in GFAP-expressing glial cells (Fig. 1C).

zDHHC enzymes have been shown to localize to the plasma membrane and/or organelle membranes (Ohno et al., 2006). To determine whether zDHHC15 is localized to the plasma membrane, we performed a surface biotinylation assay using neurons at 13 DIV (Fig. 1D). Unlike zDHHC5 or zDHHC8, which have previously been shown to localize to plasma membranes (Brigidi et al., 2015; Ohno et al., 2006), zDHHC15 was not isolated in surface fractions, suggesting it is restricted to internal membranes. Coimmunostaining with zDHHC15 and the Golgi marker giantin demonstrated that zDHHC15 is highly expressed in the somatic Golgi complex as well as in dendrites – with 67±3% of all zDHHC15-positive puncta localizing with giantin (Fig. 1E,F). The localization of zDHHC15 in the somatic Golgi of neurons is in accordance to a previous report demonstrating zDHHC15 localization to the Golgi compartment of HEK293 cells (Greaves et al., 2008) and hippocampal neurons (Levy et al., 2011).

As zDHHC15 was distributed in a punctate manner within dendrites, we determined whether zDHHC15 localized to PSD-95 and gephyrin puncta that – as demonstrated by us – are faithful excitatory and inhibitory markers, respectively (Fig. S2A-D). The density of colocalized zDHHC15 and PSD-95, and zDHHC15 and gephyrin puncta was quantified within eGFP ‘masks’ of neurons (Fig. S2E). Coimmunostaining demonstrated minimal localization of zDHHC15 at synapses (Fig. 1G). Only 18±2% of zDHHC15-positive puncta colocalized with PSD-95 and 10±2% colocalized with gephyrin (in turn 22±2% of PSD-95-positive and 23±3% of gephyrin-positive puncta colocalized with zDHHC15) (Fig. 1G,H). Although the modest colocalization of PSD-95 and zDHHC15 may appear surprising – as PSD-95 is a known substrate of zDHHC15 (Fukata et al., 2004) – zDHHC15 function is thought to be dependent on synaptic activity (Noritake et al., 2009). It is, therefore, possible that the subcellular localization of zDHHC15 is altered following changes in synaptic activity. Together, these data suggest that zDHHC15 mediates its function during early stages of neuronal development, and that its primary site of action is at Golgi compartments that are distinct from Golgi satellites associated with excitatory or inhibitory synapses.

### zDHHC15 promotes dendritic outgrowth and arborization

**Fig. 2.**
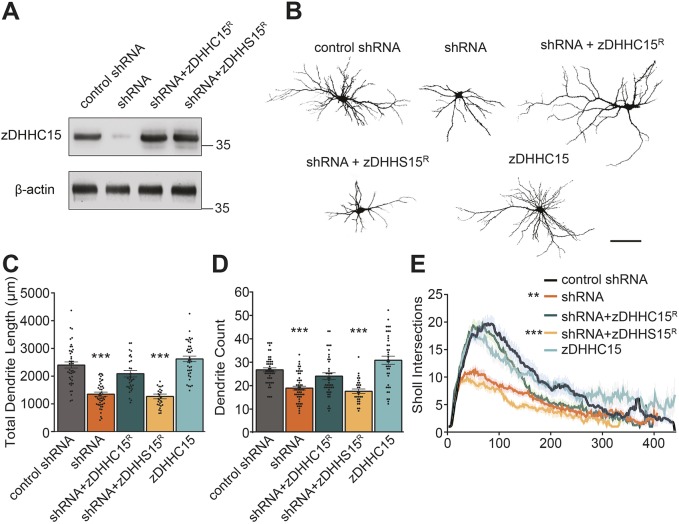
**zDHHC15 promotes dendritic growth and arborization.** (A) Western blot of rat hippocampal neurons nucleofected at 0 DIV, lysed at 4 DIV and probed with indicated antibodies. *n*=3 cultures. (B) Rat hippocampal neurons were transfected with the indicated constructs, fixed and imaged at 13 DIV. Images shown have been manually masked to remove background axons; raw images are available in Fig. S3A with an example of masking, tracing and Sholl analysis available in Fig. S3B. Scale bar: 100 µm. (C-E) Quantification of dendrite length and counts from 13 DIV rat hippocampal neurons. Knockdown of zDHHC15 (using shRNA targeting zDHHC15, referred to as shRNA) decreases dendritic length and complexity. Statistical significance was calculated for the entire Sholl profile. *n*=43 (control), 49 (shRNA), 34 (shRNA+zDHHC15), 40 (shRNA+zDHHS15), 36 (zDHHC15) neurons, three cultures. **P<0.01, ***P<0.001, one way ANOVA and Tukey's post-hoc test; mean±s.e.m.

In humans, the *ZDHHC15* gene is located on the X-chromosome and loss of *ZDHHC15* transcript has previously shown to be associated with intellectual disabilities (Mansouri et al., 2005). Based on this and the high expression of zDHHC15 protein during development, we hypothesized that zDHHC15 is important for neuronal connectivity. To test this, we generated small hairpin RNA targeting zDHHC15 (shRNA; validated in Fig. 2A) and examined whether zDHHC15 knockdown impacts neuronal morphology (see Fig. S3A,B for raw images, neuron masking, tracing and Sholl analysis). Neurons expressing zDHHC15 shRNA exhibited significantly shorter dendrites (Fig. 2B,C), decreased branch count (Fig. 2B,D) and less dendritic complexity (Fig. 2B,E) compared to neurons transfected with control shRNA. These dendritic defects were rescued in cells expressing an shRNA-resistant zDHHC15 construct (zDHHC15^R^) (Fig. 2B-E). To determine whether the palmitoylation function of zDHHC15 is important for dendritic outgrowth, cells were co-transfected with shRNA and palmitoylation-deficient zDHHC15, in which the cysteine residue of DHHC had been changed to serine (zDHHS15^R^). Although expression of zDHHS15^R^ was similar to that of zDHHC15^R^ (Fig. 2A), it was unable to rescue the knockdown phenotype, indicating that the enzymatic activity of zDHHC15 is essential for zDHHC15-mediated regulation of dendritic arbor length and complexity (Fig. 2B-E). Overexpression of zDHHC15 had no effect on dendrite length, number or complexity (Fig. 2B-E).

**Fig. 3.**
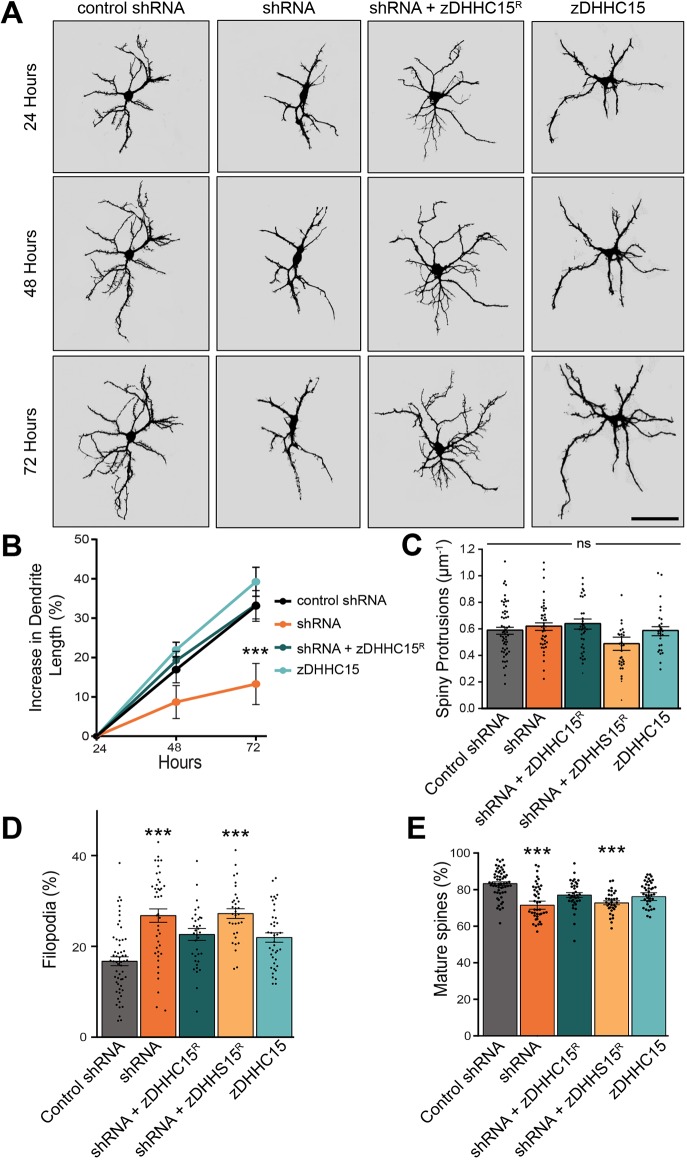
**zDHHC15 knockdown leads to inhibition of dendritic outgrowth and formation of mature spines.** (A) Representative, masked, time-lapse confocal images of neurons transfected at 10 DIV with eGFP plus the indicated constructs and imaged 24, 48 and 72 h post transfection. Raw images are available in Fig. S3C. Scale bar: 100 µm. (B) zDHHC15 knockdown inhibits dendrite growth. *n*=10 neurons, three cultures. (C-E) Hippocampal neurons at 10 DIV transfected with the indicated constructs, fixed, and imaged at 13 DIV to calculate the density (C) and type (D,E) of spiny protrusions. While there was no significant change in the density of overall protrusions (C), zDHHC15 knockdown significantly increased the proportion of filopodia (D) and reduced the proportion of mature, mushroom and stubby spines (E). *n*=56 (control), 43 (shRNA), 38 (shRNA+zDHHC15), 33 (shRNA+zDHHS15), 31 (zDHHC15) neurons, three cultures. ***P<0.001; ns, not significant; one-way ANOVA and Tukey's post-hoc test (C-E), mean±s.e.m.

To further investigate whether zDHHC15 promotes dendritic outgrowth or stabilization, neurons were imaged every 24 h for 72 h post transfection using time-lapse imaging. At 72 h post transfection, cells expressing zDHHC15 shRNA exhibited an increase of only 13±4.7% in total dendritic length, whereas dendrite length of cells expressing control shRNA, shRNA+zDHHC15^R^ or zDHHC15 alone increased by 33±3.9%, 34±4.0% or 39±6.1%, respectively (Fig. 3A,B, raw images Fig. S3C). These data indicate that zDHHC15 is required for dendritic outgrowth and not stability, because knockdown of zDHHC15 inhibited further dendritic outgrowth.

### zDHHC15 promotes spine maturation and the formation of excitatory synapses

We next determined whether zDHHC15 regulates the formation and/or maturation of spiny protrusions – including filopodia – that exhibit dynamic motility and are associated with smaller, less-mature synapses, as well as with stubby or mushroom spines, which are typically associated with large, mature synapses (Casanova et al., 2012; Marin-Padilla, 1972). Although knockdown of zDHHC15 did not impact the total number of spiny protrusions at 13–14 DIV (Fig. 3C), it resulted in a significant increase in the proportion of thin, immature protrusions, and a decrease in the proportion of mature, stubby and mushroom spines (Fig. 3D,E). Although zDHHC15^R^ rescued this knockdown phenotype, palmitoylation-defective zDHHS15^R^ did not, indicating that zDHHC15 regulates spine morphology and maturation through its palmitoylation function.

**Fig. 4.**
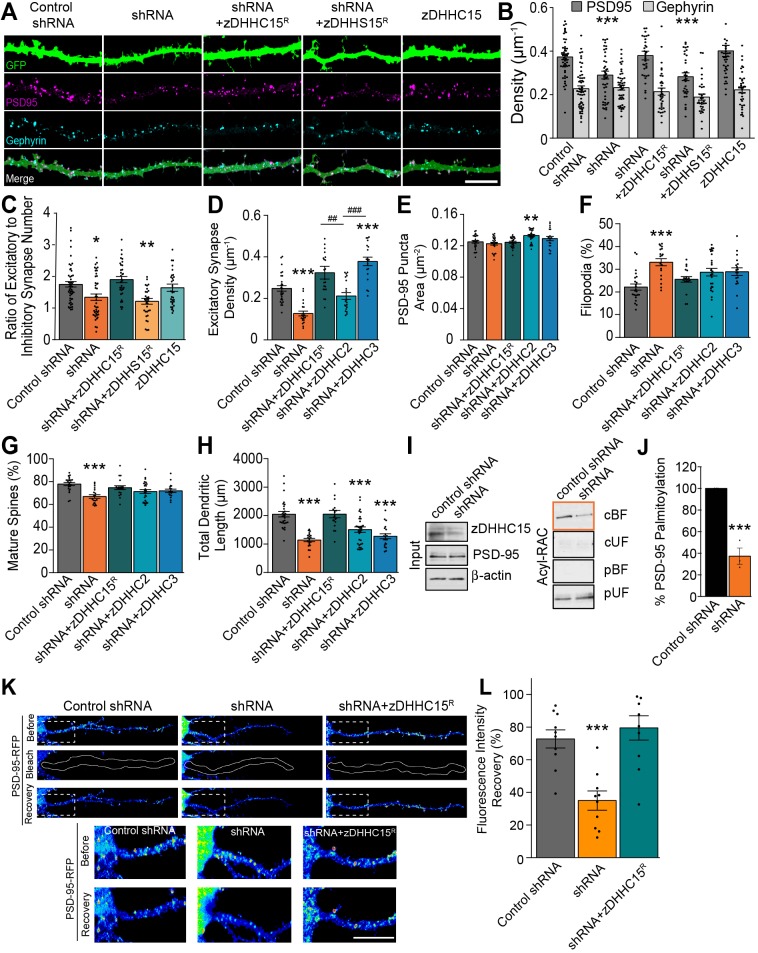
**zDHHC15 knockdown decreases excitatory synapse density in hippocampal neurons and disrupts PSD-95 trafficking into dendrites.** (A) Representative confocal images of rat hippocampal neurons at 13 DIV expressing eGFP plus the indicated constructs. Control shRNA is a scrambled form of the shRNA targeting zDHHC15 (zDHHC15 shRNA, shortened to shRNA) to ensure there are no off-target effects of the shRNA. zDHHC15^R^ is a form of zDHHC15 that has had mutations introduced to modify the sequence recognized by the targeting shRNA to make it shRNA resistant. zDHHS15^R^ is a construct wherein the catalytic cysteine has been modified to serine to reduce palmitoylation activity and is also resistant to the targeting shRNA. Immunostaining was for the excitatory post-synaptic marker, PSD-95 and the inhibitory post-synaptic marker gephyrin (masked as per Fig. S2E). Scale bar: 10 µm. (B) The density of PSD-95-positive puncta is significantly decreased in zDHHC15 knockdown neurons, while the density of gephyrin-positive puncta remains unchanged. (C) This alters the ratio of excitatory:inhibitory synapse density being formed onto zDHHC15 mutant neurons. *n*=51 (control), 43 (shRNA), 33 (shRNA+zDHHC15), 34 (shRNA+zDHHS15), 33 (zDHHC15) neurons, three cultures. (D) The zDHHC15 shRNA-mediated reduction in excitatory synapse density (density of colocalized PSD-95 and excitatory pre-synaptic marker VGlut1 puncta) is rescued by coexpression of zDHHC15, zDHHC2 or zDHHC3. (E) The area of PSD-95 puncta is unchanged in cells expressing zDHHC15 shRNA but significantly increased compared to controls in cells expressing zDHHC15 shRNA plus zDHHC2. (F,G) zDHHC15, zDHHC2 and zDHHC3 rescue zDHCH15 shRNA-mediated changes in the proportion of filopodia (F) and mature spines (G). (H) By contrast, zDHHC2 and zDHHC3 are unable to rescue zDHHC15-mediated decreases in dendritic length. *n*=25 neurons, three cultures. (I) Acyl-RAC assay from cultured neurons at 7 DIV that were nucleofected with control or zDHHC15 shRNA. Left panel (input levels): zDHHC15 was reduced by 66.25±6% in neurons that had been transfected with zDHHC15 shRNA, reflecting the efficiency of transfection. No changes in PSD-95 levels were observed. Right panel (Acyl-RAC fractions): Results from the same blot; PSD-95 palmitoylation is decreased in cultures transfected with zDHHC15 shRNA (cleaved bound fraction, cBF), low levels of non-palmitoylated PSD-95 (cUF, cleaved unbound fraction), and minimal non-specific binding to the resin (pBF, preserved bound fraction). The pUF (preserved unbound fraction) represents samples that have not been cleaved. (J) PSD-95 palmitoylation is decreased in zDHHC15 knockdown neurons. *n*=3 cultures. ***P<0.001, Student's *t*-test, mean±s.e.m. (K) Hippocampal neurons at 10 DIV were transfected with the indicated constructs plus PSD-95-RFP. Puncta of PSD-95-RFP within an entire primary dendrite branch (white outline) were photobleached at 13 DIV and fluorescence recovery was analysed 9 h after bleaching. Fluorescence recovery was decreased in cells expressing zDHHC15 shRNA (PSD-95 puncta pseudo-colored for fluorescence intensity). Magnified area of interest shown below. Scale bar: 10 µm. (L) Quantification of fluorescence recovery. *n*=10 neurons, three cultures. ***^/###^*P*<0.001, **^/##^*P*<0.01, *P<0.05, one-way ANOVA and Tukey's post-hoc test, mean±s.e.m.

As the excitatory postsynaptic protein PSD-95 can be palmitoylated by zDHHC15 (as well as by zDHHC2, zDHHC3 and zDHHC7) (Fukata et al., 2004), and because knockdown of zDHHC15 decreases the proportion of mature spines (Fig. 3D,E), we next investigated whether zDHHC15 regulates the formation and/or maintenance of synaptic connections. At 10 DIV, cultures were transfected with eGFP plus the constructs indicated in Fig. 4A, masks were generated as previously described (Fig. S2E) (Brigidi et al., 2015), and cells immunostained for PSD-95 and gephyrin. zDHHC15 knockdown led to a significant reduction in the density of puncta positive for PSD-95 but not gephyrin (Fig. 4A,B), causing an overall decrease in the ratio of excitatory to inhibitory (E:I) synapses (Fig. 4C). Of note, zDHHC15 knockdown also resulted in a decrease in the density of colocalized PSD-95 and/or VGlut1 (vesicular glutamate transporter 1, an excitatory presynaptic marker) demonstrating a reduction in excitatory synapse number, and not just PSD-95 density (Fig. 4D, Fig. S2F). While expression of wild-type zDHHC15^R^ rescued the knockdown phenotype, zDHHS15^R^ did not, indicating that the enzymatic activity of zDHHC15 is important for its regulation of excitatory synapse density (Fig. 4A-C).

As PSD-95 is also palmitoylated by zDHHC2, zDHHC3 and zDHHC7 (Fukata et al., 2004), we determined whether overexpression of these enzymes can rescue the zDHHC15 knockdown phenotype (we did not use zDHHC7 as its levels are low in the hippocampus) (https://www.proteinatlas.org/ENSG00000153786-ZDHHC7/tissue) and proved difficult to express (validation of zDHHC2 and zDHHC3 expression, Fig. S4). The zDHHC15 shRNA-mediated decrease in PSD-95 density was rescued by zDHHC3 and zDHHC15 and, to a lesser extent, zDHHC2 (Fig. 4D). Moreover, while zDHHC15 knockdown did not impact PSD-95 puncta size, coexpression of zDHHC15 shRNA with zDHHC2 enhanced the area of PSD-95 clusters by around 10%, suggesting a different mechanism of action (Fig. 4E). In concordance with zDHHC2 and zDHHC3's ability to rescue PSD-95 density, expression of these proteins rescued spine maturation (Fig. 4F,G). However, in contrast to the ability of zDHHC2 and zDHHC3 to rescue synapse density and spine maturation, neither zDHHC2 nor zDHHC3 rescued zDHHC15 knockdown-mediated changes regarding the dendritic length (Fig. 4H). These results demonstrate that zDHHC15 knockdown decreases excitatory synapse density and outgrowth, and that the pathway regulating excitatory synapse density is independent of the pathway involved in dendritic length and complexity.

### zDHHC15 promotes the trafficking of PSD-95 into dendrites

As zDHHC15 is known to palmitoylate PSD-95, and because excitatory synapse density is reduced following zDHHC15 knockdown, we tested whether PSD-95 palmitoylation is decreased in zDHHC15 knockdown cells. Hippocampal neurons were nucleofected with zDHHC15 shRNA resulting in a 58±3.8% efficiency of transfection (investigated by counting the eGFP-positive transfected cells in each culture before lysis). By using the acyl-RAC assay, we demonstrated a 62.5±7.54% reduction in PSD-95 palmitoylation in primary neuron cultures transfected with zDHHC15 shRNA compared to control shRNA, with no change in total PSD-95 levels (Fig. 4I,J).

Previous work has demonstrated that the N-terminal palmitoylation motif of PSD-95 is necessary for targeting of PSD-95 into dendrites (El-Husseini Ael et al., 2001), as well as its association with vesiculotubular structures that traffic PSD-95 to synapses (El-Husseini et al., 2000). To determine whether zDHHC15 is specifically involved in the targeting and trafficking of PSD-95, hippocampal neurons were transfected with PSD-95-RFP plus the constructs indicated in Fig. 4K. All PSD-95 puncta within an entire dendrite were photobleached, and fluorescent recovery within the photobleached dendrite was measured 9 h after photobleaching (Fig. 4K,L). While neurons transfected with control shRNA exhibited 73±7% recovery of PSD-95-RFP fluorescence, neurons transfected with zDHHC15 shRNA exhibited only 35±6% recovery. This knockdown phenotype was rescued by co-transfecting neurons with zDHHC15^R^ (80±8% recovery) (Fig. 4K,L). In support of the fact that zDHHC15 regulates trafficking of PSD-95 into dendrites, PSD-95-RFP levels were significantly higher in the soma of neurons expressing shRNA targeting zDHHC15 (referred to in the figure as shRNA) (Fig. 4K).

Our data suggest that the decrease in excitatory synapse density within zDHHC15 knockdown neurons is due to decreased PSD-95 trafficking into dendrites. While the synapse density phenotype in knockdown cells could be rescued by zDHHC15, zDHHC3 and zDHHC2, all of which mediate PSD-95 palmitoylation (Fukata et al., 2004), the rescue with either zDHHC3 or zDHHC15 was significantly more robust than the rescue with zDHHC2. As zDHHC3 and zDHHC15 are both localized to the somatic Golgi (Greaves et al., 2011; Noritake et al., 2009), it is likely that these two enzymes regulate excitatory synapse density by palmitoylating PSD-95 and promoting the trafficking of PSD-95 into dendrites. In contrast, zDHHC2 is localized to dendritic compartments (Greaves et al., 2011; Noritake et al., 2009), and has been shown to mediate PSD-95 palmitoylation and to promote its clustering at synapses (Noritake et al., 2009).

Together, our data demonstrate a role for zDHHC15 in the regulation of dendritic outgrowth, and the formation and maturation of glutamatergic synapses. This is of particular interest, given the association between zDHHC15 and X-linked intellectual disability (Mansouri et al., 2005; Piton et al., 2013), and the fact that disruptions in dendrite outgrowth and synapse function are one of the most-consistent hallmarks of intellectual disability, observed in both postmortem brain sections and mouse models of intellectual disability (Casanova et al., 2012; Huttenlocher, 1974; Jiang et al., 1998; Marin-Padilla, 1972; Purpura, 1974; Swann et al., 2000).

## MATERIALS AND METHODS

### Contact for reagent and resource sharing

Further information and requests for reagents may be directed to and will be fulfilled by the corresponding author S.X.B.

### Antibodies

Primary antibodies used were: anti-zDHHC15 (Abcam, Cambridge, MA, #ab121203, 1:250, western blot), anti-zDHHC15 (Thermo Scientific, Waltham, MA, #PA39327, immunofluorescence, 1:100), anti-Flag (Sigma Aldrich, St Louis, MO, #F3165, 1:1000), anti-Myc (Origene, Rockville, MD, #TA150121, 1:1000), anti-β-actin (Novus Biologicals, Centennial, CO, #NB600-503, 1:1000), anti-PSD-95 (Abcam, Cambridge, MA, #ab2723, 1:500), anti-gephyrin (Synaptic Systems, Göttingen, Germany #147011, 1:500), anti-GAD65 (Synaptic Systems, Göttingen, Germany #198104, 1:500), anti-GFAP (Synaptic Systems, Göttingen, Germany #173004, 1:400), anti-MAP2 (Millipore, Burlington, MA, MB3418, 1:2000), anti-giantin (Synaptic Systems, Göttingen, Germany, #263004, 1:500), anti-GM130 (BD Biosciences, Franklin Lakes, NJ, #610822, 1:200). Secondary antibodies from Life Technologies (Carlsbad, CA,) used: (1:500), anti-mouse AF568 IgG2a (#A21134), anti-mouse AF647 IgG1 (#21240), anti-rabbit AF488 (#A11008), anti-rabbit AF568 (#A11011), anti-guinea pig AF633 (#A21105). HRP conjugated secondary antibodies were obtained from Bio-Rad (Hercules, CA): goat anti-mouse #170-6516 and goat anti-rabbit (#170-6515, 1:300). DAPI was used for nuclear staining (Life Technologies, Carlsbad, CA, #D1306, 1:1000).

### Plasmids and primers

The rat Myc-DDK tagged zDHHC15 ORF construct was obtained from Origene (Rockville, MD, #RR212342). The shRNA target sequence that gave maxium knockdown efficiency of zDHHC15 was 5′-GGTTCAATCTTGGCTTCATCA-3′ and was cloned into the pLL3.7 vector (a gift from Luk Parijs; Addgene plasmid #11795) using the following sense and anti-sense sequences based on previous cloning experiments in pLL3.7 using XhoI and HpaI sites (Rubinson et al., 2003): Sense: 5′-TGGTTCAATCTTGGCTTCATCATTCAAGAGATGATGAAGCCAAGATTGAACCTTTTTTC-3′ and antisense: 5′-TCGAGAAAAAAGGTTCAATCTTGGCTTCATCATCTCTTGAATGATGAAGCCAAGATTGAACCA-3′. This plasmid is referred to as shRNA in all figures. To generate a scrambled shRNA construct to control for potential off-target effects of shRNA (control shRNA), we used the following sense and anti-sense sequence: Sense: 5′-TGAAGTTCGTACTTATCTCCGTTTCAAGAGAACGGAGATAAGTACGAACTTCTTTTTTC-3′ and antisense: 5′-TCGAGAAAAAAGAAGTTCGTACTTATCTCCGTTCTCTTGAAACGGAGATAAGTACGAACTTCA-3′. To generate an shRNA-resistant form of zDHHC15 (zDHHC15^R^), site-directed mutagenesis (Agilent Technologies, Santa Clara, CA: QuikChange II XL Site-Directed Mutagenesis Kit, 200521) of the zDHHC15 ORF sequence GGTTCAATCTTGGCTTCATCA into GGTTTAATCTAGGATTCATAA was carried out by using the following forward and reverse primers: Forward 5′-CAGAGAAAAATGGGTTTAATCTAGGATTCATAAAGAATATTCAG-3′ and Reverse 5′-CTGAATATTCTTTATGAATCCTAGATTAAACCCATTTTTCTCTG-3′ (underlined residues indicate amino acid codons where nucleotide bases were mutated). zDHHS15^R^ was made by using the zDHHC15^R^ template with the following forward and reverse primers: Forward 5′-AAATGGACCATCATTCCCCATGGGTTAAT-3′ and Reverse 5′-ATTAACCCATGGGGAATGATGGTCCATTT-3′.

### Experimental model and subject details

#### Animals

All experimental procedures and housing conditions were approved by the UBC Animal Care Committee and were in accordance with the Canadian Council on Animal Care (CCAC) guidelines.

### Primary culture from Sprague-Dawley rats

Hippocampi from embryonic day 18 (E18) Sprague-Dawley rats (*Rattus norvegicus*) of either sex were prepared and plated on 18 mm Marienfeld (Lauda-Königshofen, Germany) coverslips for 12-well plates or directly onto the plastic of 6-well plates at a density of 130 cells/mm^2^. Coverslips had previously been treated with concentrated nitric acid (Sigma Aldrich, St Louis, MO) overnight, rinsed several times with milliQ water, then sterilized with high heat (>250°C overnight). Coverslips were then placed in the 12-well plate, UV sterilized for 30 min, and coated with 0.5 mg/mL poly-L-lysine hydrobromide mixed with borate buffer. The plates were covered with aluminium foil and left in a biosafety cabinet overnight. The following day, coverslips were rinsed 3× with autoclaved milliQ water and plating medium (84.75 ml MEM with Earle's BSS, 10 ml FBS, 2.25 ml of 20% glucose, 1 ml sodium pyruvate, 1 ml 100×GlutaMax, 1 ml 100×Pen/Strep) was added (1 ml per 12 well, 2 ml per 6 well). Plates were then placed in the 5% CO_2_ incubator at 37°C overnight.

For dissection, timed-pregnant Sprague-Dawley rats (*Rattus norvegicus*, Jackson Laboratories, Sacramento, CA) were euthanized using CO_2_ and cervical dislocation. Pups were harvested into 150-mm Petri dishes and decapitated, and the brains were then dissected out and placed in pre-chilled HBSS on ice. Hippocampi were dissected and placed in a 15 ml conical tube with 10 ml of fresh pre-warmed (37°C) HBSS. Once hippocampi settled, supernatant was removed and replaced with 10 ml of fresh pre-warmed HBSS for a total of three rinses. After the final rinse, 4.5 ml of fresh, pre-warmed HBSS was added to the hippocampi with 0.5 ml of 2.5% trypsin and incubated in a 37°C water bath for 20 min with gentle agitation every 5 min. In the final 3 min, 1% DNase was added and gently agitated to ensure mixture. Hippocampi were then rinsed 3× with fresh, prewarmed HBSS and, after the final wash, ∼1 ml of HBSS was used to transfer hippocampi to a 60-mm dish for trituration. The total volume following trituration was brought up to 5 ml and cell density was determined using a hemocytometer to plate at the desired density. At 3-4 h after plating, plating medium was aspirated and replaced with pre-warmed (37°C) maintenance media (97 ml Neurobasal medium, 2 ml 50× B-27 supplement, 1 ml 100×GlutaMax, 1 ml 100× Pen/Strep). Maintenance medium was renewed with fresh maintenance medium 2–3 days following plating.

### Method details

#### Transfection (primary hippocampal cultures/HEK293T cells)

Primary hippocampal cultures were transfected at 9–11 DIV by using Lipofectamine 2000 (Invitrogen, Carlsbad, CA) according to the manufacturer's recommendations. Briefly, for 12-well-plates, two aliquots of 25 μl Opti-Mem (Gibco, Thermo Fisher Scientific, Waltham, MA) were prepared. To one aliquot, 1–3 μg of total plasmid DNA was added. To the other aliquot, 1 μl of Lipofectamine 2000 was added and allowed to mix for 5 min. After this period, aliquots were combined and incubated for 20 min, then added drop by drop to the 12-well plates. Cells were then live imaged (10–13 DIV) or fixed (13 DIV) for subsequent experiments. HEK293T cells were transfected with Lipofectamine 2000 (Invitrogen) at 70% confluency at a ratio of 3:1 (shRNA or scrambled shRNA to target plasmid) and incubated for 48–72 h before harvesting for biochemical analysis. For HEK293T cells, 150 µl Opti-Mem were used with 6 µl Lipofectamine 2000.

Nucleofections for 6-well plate biochemical assays were performed immediately before plating at 0 DIV by using an Amaxa Nucleofection Kit (Lonza, Basel, Switzerland, VPG-1003) according to the manufacturer's optimized protocol (Number 101, program G-13), and were used for experiments at either 4 DIV (Fig. 2A) or 7 DIV (Fig. 4I). Briefly, hippocampi were dissociated as above and ∼500,000 neurons isolated for nucleofection. Neurons were mixed with 100 μl Ingenio electroporation solution (Mirus Bio, Madison, WI) and cDNA constructs (∼3 μg in total) were added to the solution. The solution was transferred to an Amaxa Nucleofection cuvette and inserted into the Amaxa Nucleofector with program G-013. Following electroporation, the solution was plated into a 6-well dish for future biochemical experiments.

#### Immunocytochemistry

Immunocytochemistry experiments were performed as previously reported (Sun and Bamji, 2011). Briefly, cells were fixed for 10 min in a pre-warmed (37°C) 4% paraformaldehyde–sucrose solution. Cells were then washed in phosphate buffered saline (PBS) and treated with 0.1% Triton X-100 in PBS for 10 min at room temperature, washed in PBS, and blocked for 1 h at room temperature with 10% goat serum in PBS. Cells were then incubated in 1% goat serum in PBS with primary antibodies overnight at 4°C. Subsequently, cells were washed 3× in PBS for 10 min each, incubated in secondary antibodies in 1% goat serum in PBS for 1 h at RT, washed again 3× in PBS and mounted on microscope slides by using Prolong Gold (Molecular Probes, Thermo Fisher Scientific, Waltham, MA). For DAPI staining, a 1:100 dilution from a 5 mg/ml stock was applied after all other staining procedures for 5 min. Cells were then washed 3× in PBS and then mounted as above.

#### Biotinylation

For surface biotinylation assays, neurons in 6-well plates were washed with ice-cold PBS pH 8 supplemented with 0.1 mM CaCl_2_ and 1 mM MgCl_2_ (PBS-CM) and then incubated for 30 min with 0.5 mg/ml NHS-SS-Biotin (Thermo Fisher Scientific, Waltham, MA) in ice-cold PBS-CM at 4°C with gentle rocking. After incubation, cells were washed once with PBS-CM; the unbound biotin was then quenched during two 8 min incubations with quenching buffer (20 mM glycine in PBS-CM). Lysis was performed by mechanical scraping in lysis buffer (1% IGEPAL-CA630 and 1 mM PMSF with Roche complete protease inhibitor tablet) and followed by centrifugation at 500 ***g*** for 5 min at 4°C. Samples were then vortexed, run 3–4 times through a 26½ gauge syringe, and nutated at 4°C (VWR Nutating Mixer, 20° fixed tilt angle with a mixing speed of 24 rpm) for 30 min. After nutation, samples were centrifuged at 16,100 ***g*** for 30 min at 4°C to clear the lysate. Protein content in the cell lysate was then quantified using a BCA Assay Kit (Thermo Fisher Scientific, Waltham, MA) as per the manufacturer's instructions. Whole-cell lysate (10 µg) was then combined with SDS-sample buffer (50 mM Tris-HCl, 2% SDS, 10% glycerol, 14.5 mM EDTA and 0.02% Bromophenol Blue with 1% β-mercaptoethanol), boiled for 5 min at 95°C and stored at −20°C as the input sample. Of the remaining protein sample, 100–200 µg was added to 50 µl of a 50% slurry of NeutrAvidin-conjugated agarose beads (Thermo Fisher Scientific, Waltham, MA) that had been pre-washed three times in lysis buffer. Each sample was then brought to a total volume of 500 µl with lysis buffer and nutated (VWR Nutating Mixer, 20° fixed tilt angle with a mixing speed of 24 rpm) overnight at 4°C. The following day, beads were pelleted and washed seven times by centrifugation at 500 ***g*** for 3 min, each time discarding the supernatant. Beads were eluted using 40 µl of SDS-sample buffer supplemented with 100 mM DTT and samples were boiled at 90°C for 5 min followed by western blotting with whole-cell lysates.

#### Acyl-RAC (palmitoylation) assay

For Acyl-RAC assays, we followed the manufacturer's protocol from the CAPTUREome S-palmitoylated protein kit (Badrilla, Leeds, UK), with minor changes. We optimized the protocol for our experiments by adding DNase to the lysed-cell solution. Additionally, we measured protein concentration (BCA Assay, Thermo Fisher Scientific, Waltham, MA) after dissolving the precipitated protein and prior to separating the lysate into experimental (Thioester Cleavage Reagent) and negative control (Acyl Preservation Reagent) samples to ensure accurate loading of equal protein concentrations.

#### Western blot analysis

Western blotting was performed as previously described (Sun and Bamji, 2011). Primary hippocampal neurons from Sprague-Dawley rats, HEK293T cells, or brain regions from mouse (i.e. cortex or cerebellum) were lysed at the time points indicated in figures (Fig. 1A, mouse brain regions, E17, P10, P90; Fig. 1D, hippocampal neurons, 13 DIV; Fig. 2A, Fig. S4, hippocampal neurons, 4 DIV; Fig. 4I, hippocampal neurons, 7 DIV; Fig. S1A, HEK293T cells, 95–100% confluency) in ice-cold RIPA buffer or lysis buffer (1% IGEPAL CA-630, 50 mM Tris-HCl-pH 7.5, 150 mM NaCl, 10% glycerol) supplemented with protease inhibitor cocktail solution at 4°C on a nutator (VWR Nutating Mixer) for 2 h at 20° fixed tilt and 24 rpm. Lysates were cleared by benchtop centrifugation (16,000 ***g***) for 30 min at 4°C and the supernatant was mixed with 5×SDS dye for SDS-PAGE. Proteins were separated on either a 10% resolving gel or a 4–20% gradient gel, transferred onto a PVDF membrane, blocked in 3% BSA (in TBST) and were then immunoblotted for were then immunoblotted for zDHHC15 (Fig. 1A,D, Fig. 2A, Fig. 4I; Fig. S1A), β-actin (Fig. 1A, Fig. 2A, Fig. 4I; Fig. S1A, Fig. S4), zDHHC5 (Fig. 1D), zDHHC8 (Fig. 1D), PSD-95 (Fig. 4I) or Myc (Fig. S1A, Fig. S4) (prepared in 0.3% BSA in TBST) and visualized using enhanced chemiluminiscence reagent (Merck Millipore, #WBKLS0500) on a Li-Cor C-Digit Blot Scanner (LI-COR, Lincoln, NE). Blots were quantified using ImageJ software (NIH, Bethesda, Maryland).

#### Imaging

Fixed and live neurons were imaged by using an inverted Olympus (Richmond Hill, ONT, Canada) Fluoview 1000 (FV1000) confocal microscope. Imaging of synapses and colocalization studies utilized the 60×/1.42 Oil Plan-Apochromat objective while dendritic length measurements utilized the 20×/0.75 Oil Plan-Apochromat objective. MatTek glass-bottomed culture dishes (P35G-1.5-14-C, MatTek Corporation, Ashland, MA) were used to perform live imaging of dendrite growth of neurons in a chamber maintained at 37°C. eGFP-transfected cells with different experimental conditions were imaged in HEPES buffer (140 mM NaCl, 1.3 mM CaCl2, 1.3 mM MgCl2, 2.4 mM K2HPO4, 25 mM HEPES pH 7.4) every 24 h post transfection up to 72 h to ensure cell viability. Levels and contrast of confocal images were moderately adjusted in Photoshop CS6 software (Adobe Systems, San Jose, CA) by using scientifically acceptable procedures.

### Fluorescence recovery after photobleaching

Fluorescence recovery after photobleaching (FRAP) experiments were performed as previously described (Brigidi et al., 2014, 2015) but updated for use on an Olympus (Richmond Hill, ONT, Canada) FV3000 microscope. Briefly, cells transfected with eGFP and PSD-95 were identified and up to four dendritic branches of the cell that were clear of other obstructions and relatively unbranched were photobleached from the soma to the end of the dendrite. Cell positions were recorded using the motorized stage and ‘positions’ function of the FV3000, and re-imaged 9 h after bleaching. Fluorescence recovery was quantified using processing software within the Olympus FV3000 and then analyzed in Prism software (Graphpad, La Jolla, CA).

### Image analysis and quantification

#### Dendrite length and Sholl analysis

Neurons at 10–11 DIV were transfected and fixed at DIV 13–14. To measure dendrite lengths, neurons were identified through eGFP fluorescence and imaged at a magnification of 20×. Images were processed using Adobe Photoshop to remove axons. Images were converted to 8-bit and then exported into the ImageJ tool-NeuronJ (Meijering et al., 2004) to trace the dendritic length. Sholl analysis (dendritic complexity analysis) was performed on the same 20× images that were first thresholded and then processed using the ‘Sholl analysis’ plugin from ImageJ (Ferreira et al., 2014) with settings to create a step size of 2 µm and an ending radius entered as 'NaN' (not a number) to enable sampling of the entire image to ensure the entire dendritic tree was captured; see Fig. S3B. Data were imported into Microsoft Excel to create an average Sholl profile for each condition. For all images, the scale for ImageJ was set using the raw data from the Olympus FV1000/FV3000 dimensions to determine µm per pixel.

#### Spine analysis

Dendritic spines were classified by using the default values and analysis from NeuronStudio (version 0.9.92) adapted from Rodriguez et al. (2006), which uses a classification scheme based on the head to neck diameter ratio, length to head diameter ratio and the head diameter. For data analysis, stubby and mushroom-type spines were classified as mature spines, whereas filopodial and/or thin protrusions were considered as immature spines.

#### Synapse density, puncta size analysis and colocalization

Transfected neurons were immunostained for PSD-95 (as a marker of excitatory synapses) and gephyrin (as a marker for inhibitory synapses). Synapses within eGFP-transfected neurons were identified by making a neuronal ‘mask' created from the GFP-cell fill, imaged at 60× magnification, in Adobe Photoshop CS6 or CC2017 (Adobe Systems, San Jose, CA). The mask was devoid of cell soma and axon. We used a magic wand tool (non-contiguous, tolerance 30) to select a black background and this cell-fill GFP mask was applied onto PSD-95-positive and/or gephyrin-positive synapse marker channels to exclude synapses from other untransfected neurons and only examine synapses that had formed on mutant cells. These masks from synapse channels were then manually thresholded to make binary images and the total number of synaptic puncta was quantified using a macro in order to define synaptic puncta as having a size between 0.05 µm^2^ and 3 µm^2^. To obtain synapse density, the total number of puncta was divided by the length of the masked dendrite (see Fig. S1B). For the puncta size measurements, masked images were subjectively thresholded and PSD-95-positive puncta were measured for Feret's diameter, and then averaged for each image under each condition. Masked images were then thresholded, and the ‘Colocalization’ plugin (https://imagej.nih.gov/ij/plugins/colocalization.html) was used in a custom macro when applying the ‘Analyze Particles’ function of ImageJ (NIH, Bethesda, MA) to determine colocalized points from separate channels.

### Statistical analysis

For all experiments, data represent the mean±s.e.m. Statistical significance was measured using ANOVA with Tukey's multiple comparison analysis in Graphpad Prism v6.01 (Graphpad, La Jolla, CA). For all imaging experiments, the *n* value is the number of cells used per condition, for at least three independent cultures. Statistical significance is assumed when α<0.05, unless mentioned otherwise (Figs 3E,F and 4F,G, where the α-value is made more-stringent to 0.001). Significance is indicated when *^/#^*P*<0.05, **^/##^*P*<0.01 or ***^/###^*P*<0.001 using one-way ANOVA, Tukey's multiple comparison test with mean ±s.e.m., and determined by Prism software. All figures were generated by using Adobe Illustrator CS6 or CC2018 software in conjunction with Adobe Photoshop CS6 or CC2017 (Adobe Systems, San Jose, CA). Statistical significances (*) indicate significant differences compared with the control; significance within different rescue constructs is indicated by #.

## Supplementary Material

Supplementary information
