# Supplementary material for: Regulation of dendrite morphology and excitatory synapse formation by zDHHC15
Source: J Cell Sci. 2019 Jul 5;132(13):jcs230052. doi: 10.1242/jcs.230052 (PMC6633394; doi:10.1242/jcs.230052)
Supplement: Supplementary information [file joces-132-230052-s1.pdf]

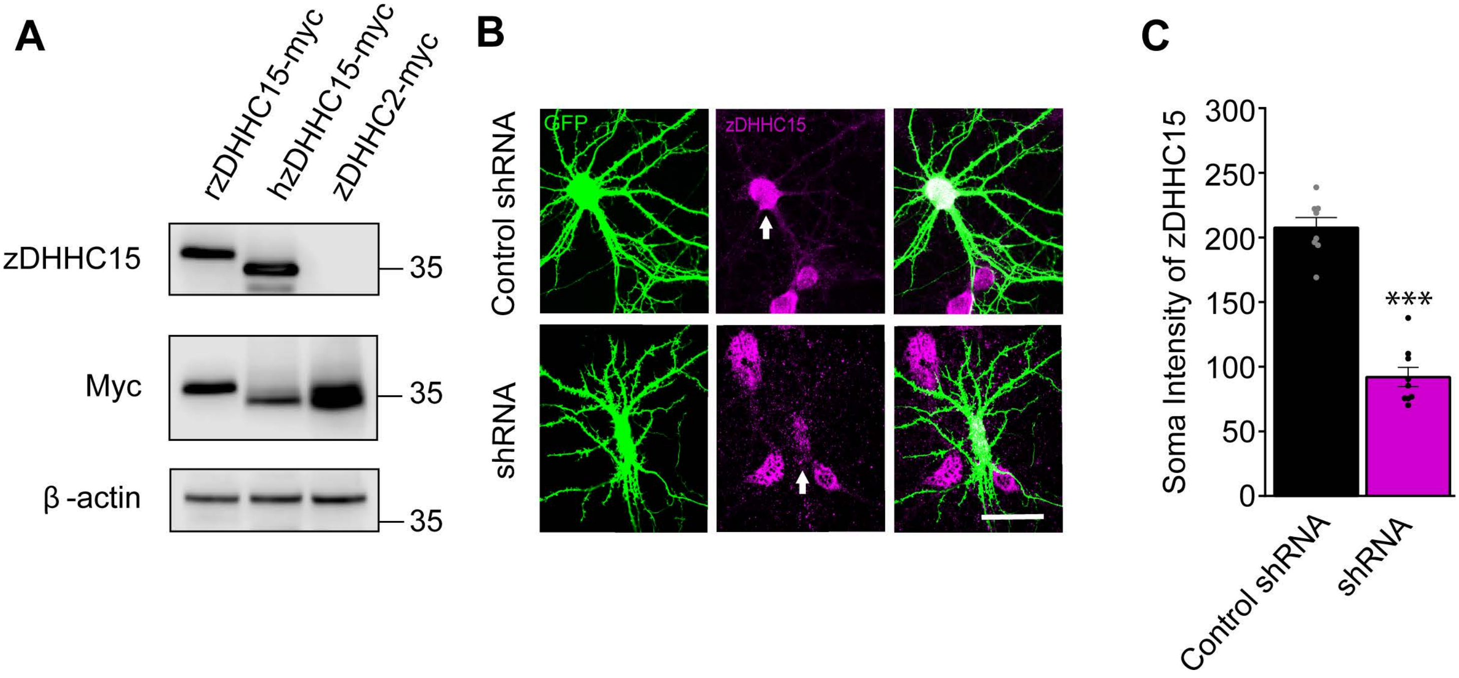

Figure S1: zDHHC15 antibody validation

(A) HEK293T cells were transfected with myc-tagged rat zDHHC15 (rzDHHC15), human zDHHC15 (hzDHHC15) or zDHHC2, the closest paralog of zDHHC15. Lysates were separated by SDS-Page and blots probed with the indicated antibodies, demonstrating the specificity of zDHHC15 antibody. Tagged-rzDHHC15 is calculated to run at 36-38kDa while -hzDHHC15 at 33-35kDa. (B) Representative confocal images of 13 DIV cultured hippocampal neurons transfected with control shRNA or zDHHC15 shRNA. Immunostaining with zDHHC15 antibody reveals decrease in immunofluorescence signal in neurons transfected with zDHHC15 shRNA, quantified in (C). Scale bar = 50μm. N = 8 neurons, 3 cultures.

Journal of Cell Science • Supplementary information

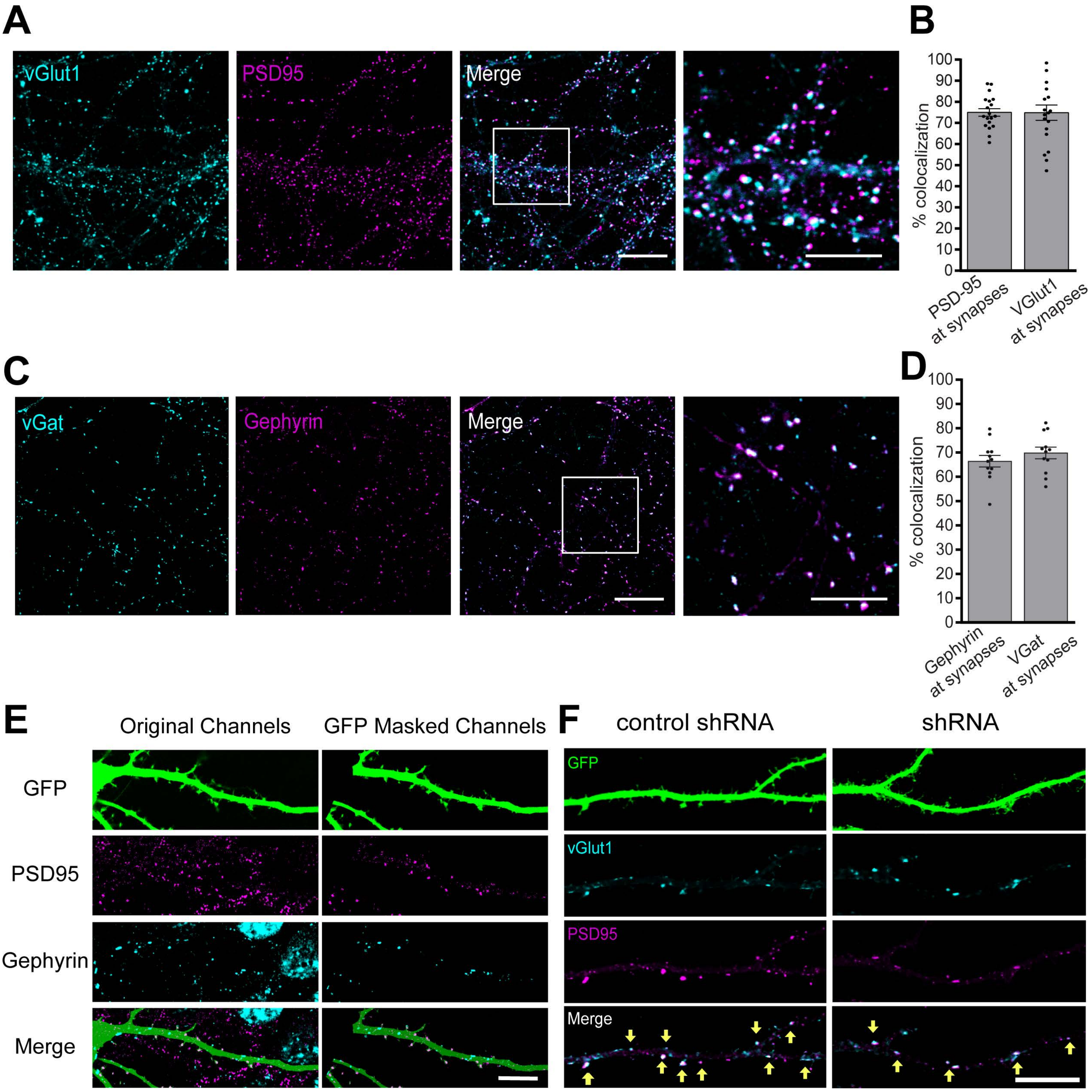
A
vGlut1
PSD95
Merge
B
% colocalization
100
90
80
70
60
50
40
30
20
10
0
PSD-95 at synapses
VGlut1 at synapses
C
vGat
Gephyrin
Merge
D
% colocalization
100
90
80
70
60
50
40
30
20
10
0
Gephyrin at synapses
VGat at synapses
E
Original Channels
GFP Masked Channels
GFP
PSD95
Gephyrin
Merge
F
control shRNA
shRNA
GFP
vGlut1
PSD95
Merge

Figure S2: Validation of synaptic markers and zDHHC15 shRNA-mediated changes in excitatory synapse density (A-D) Confocal images of 14 DIV neurons fixed and immunostained with antibodies against PSD95 and VGlut1 (A,B) or Gephyrin and VGAT (C,D) demonstrate up to 75% colocaiization indicating that Gephyrin and PSD95 are faithful markers of synapses at this timepoint. The percentage of PSD-95 at excitatory synapses was quantified by the number of PSD95/VGlut colocalized puncta divided by the total number of PSD-95 puncta(75 ± 2%). The percentage of Gephyrin puncta at inhibitory synapses was quantified by the number of Gephyrin/ VGat colocalized puncta divided by the total number of Gephyrin puncta ((67 ± 2%). (E) Illustration of masking procedure to identify and quantify immunostaining within eGFP transfected cells. Axons, background, and cell bodies were manually removed using Adobe Photoshop CS5/6 and the remaining eGFP masks overlaid on immunostained images (PSD95 and Gephyrin in this example). Raw images are shown on the left and immunostained proteins only within the eGFP mask are shown on the right. (F) Representative masked images of neurons transfected with eGFP plus either scrambled or zDHHC15 shRNA and immunostained for PSD-95 and VGlut1 demonstrate a significant reduction in the density of excitatory synapses (colocalization of PSD-95and VGlut1) as shown in Fig. 4D. Scale bars = 20µm (A, C) and 10µm (E, F).

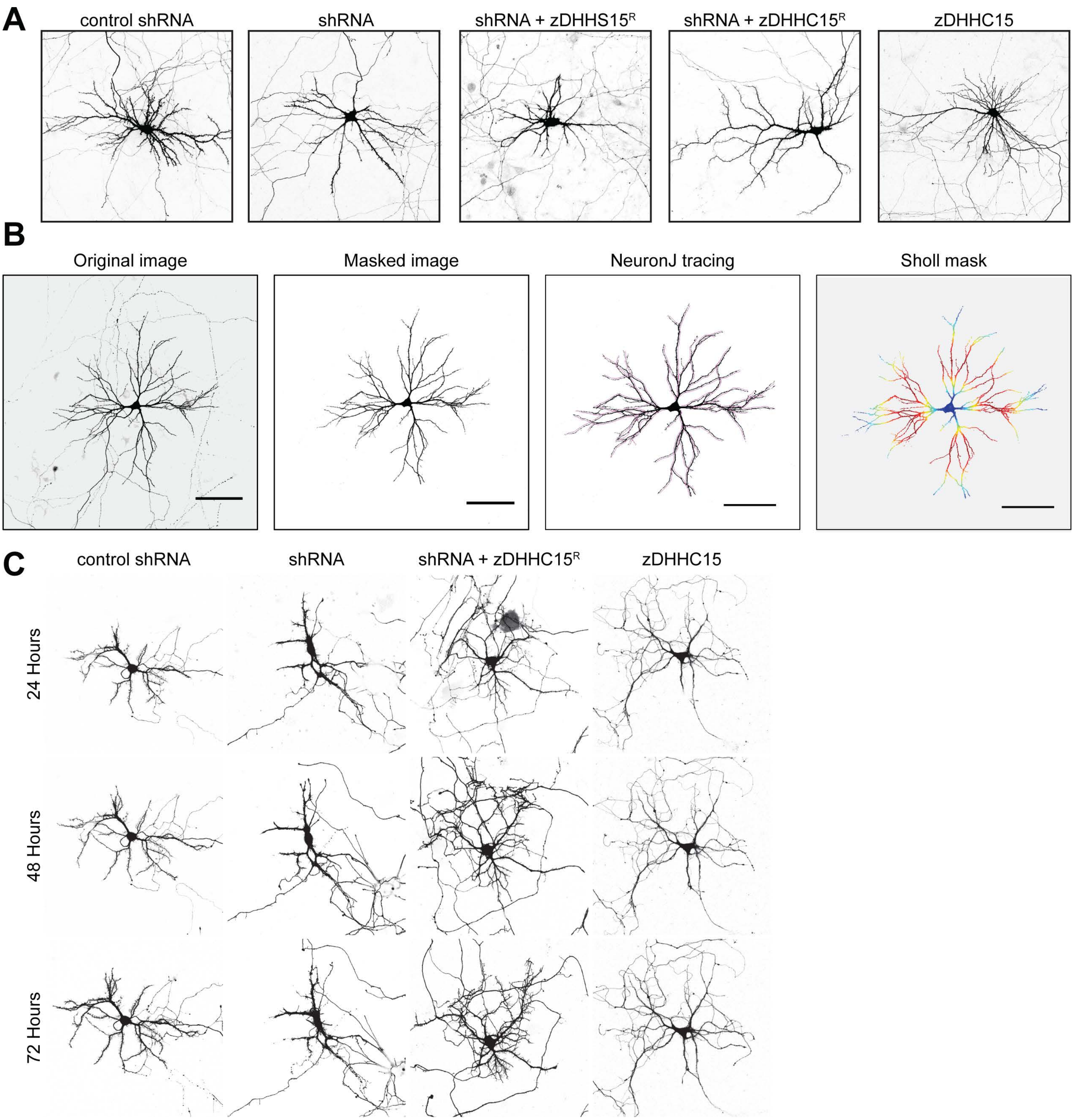

Journal of Cell Science • Supplementary information

Figure S3: Raw Images and Masking Procedures for Measuring Dendritic Length (A) Raw images of 13 DIV neurons transfected with eGFP plus indicated constructs corresponding to Figure 2B. (B) Masking procedures for neuron morphology. Raw confocal images were manually masked, removing the background and axons using Adobe Photoshop CS5/CS6 to allow quantification of only dendrites of transfected neurons. Tracing of neurons and dendritic complexity (Sholl) profiles were obtained using seperate plugins within ImageJ (Tracing - NeuronJ, Complexity - Sholl Analysis). (C) Raw images of 10 DIV neurons transfected with GFP plus indicated constructs live-imaged until 13 DIV corresponding to Figure 3A.

Journal of Cell Science • Supplementary information

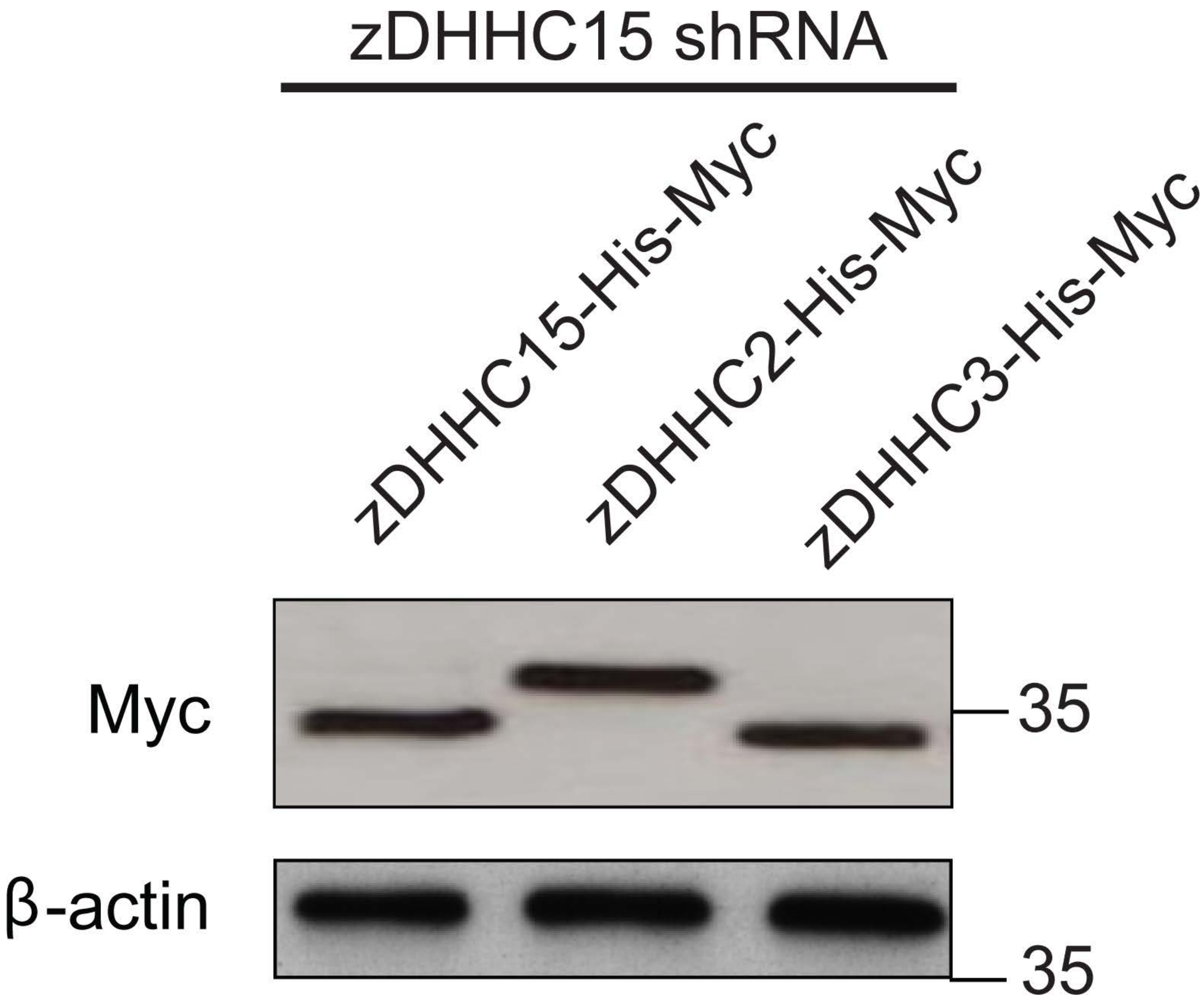

Figure S4: Expression analysis of different constructs in hippocampal neurons. Rat hippocampal neurons were nucleofected with the indicated myc-tagged constructs at the time of plating, neurons lysed at 4 DIV, and probed for indicated antibodies. The expression of zDHHC2, zDHHC3 and zDHHC15 in a zDHHC15 knockdown back-ground were similar. N = 3 cultures.
